# Efficacy and safety of maribavir in Japanese patients with post-transplant cytomegalovirus infections: a phase III study

**DOI:** 10.1007/s12185-026-04220-3

**Published:** 2026-06-12

**Authors:** Yoshinobu Kanda, Katsuto Takenaka, Tatsu Tanabe, Kazuya Omoto, Kei Kamimura, Kentaro Kudou, Masato Kaneko

**Affiliations:** 1 Division of Hematology, Jichi Medical University, Shimotsuke, Tochigi Japan; 2 Department of Hematology, Clinical Immunology and Infectious Diseases, Ehime University, Matsuyama, Ehime Japan; 3 Department of Kidney Transplant Surgery, Sapporo City General Hospital, Sapporo, Hokkaido Japan; 4 Yochomachi Clinic, Tokyo, Japan; 5 Takeda Pharmaceutical Company Limited, 1-1, Doshomachi 4-Chome, Chuo-Ku, Osaka, Japan

**Keywords:** Antiviral therapy, Cytomegalovirus, Maribavir, Transplant

## Abstract

The efficacy and safety of maribavir, an oral benzimidazole nucleoside that inhibits viral kinase UL97, were evaluated in hematopoietic stem cell transplant (HSCT) or solid organ transplant (SOT) recipients with cytomegalovirus (CMV) infection. This phase III, multicenter, open-label, single-arm, interventional study (ClinicalTrials.gov: NCT05137717; Japan Registry of Clinical Trials: jRCT2021210056) investigated oral maribavir 400 mg twice daily for 8 weeks. Japanese HSCT/SOT recipients aged ≥ 16 years with symptomatic or asymptomatic CMV infection (including those resistant/refractory to ganciclovir, valganciclovir, or foscarnet) and without central nervous system CMV tissue-invasive disease or CMV retinitis were enrolled. Primary endpoints were confirmed CMV viremia clearance at week 8 for efficacy and treatment-emergent adverse events (TEAEs) for safety. Among 61 patients enrolled across 22 sites, 41 patients (median age 56.0 years; 36/41 [87.8%] were HSCT recipients) received ≥ 1 maribavir dose. At week 8, twenty-eight patients (28/41, 68.3%) achieved confirmed CMV viremia clearance. Overall, 36 patients (36/41, 87.8%) had ≥ 1 TEAE, including nausea (10/41, 24.2%), anemia (7/41, 17.1%), pyrexia (6/41, 14.6%), and headache (6/41, 14.6%). Two deaths were deemed unrelated to maribavir. In conclusion, maribavir was effective in achieving CMV viremia clearance, with an acceptable safety profile and tolerability, in Japanese HSCT or SOT recipients with CMV infection.

## Introduction

Cytomegalovirus (CMV), a member of the Herpesviridae family [1], is estimated to be present in 83% of the global population [2]. However, in most cases, CMV exists as a subclinical latent infection [3]. In infections that are active, symptoms include fever and malaise, and in the case of invasive disease, colitis, pneumonia, and retinitis [4]. Transplant recipients are at risk of reactivation of CMV due to immunosuppressant therapies or a primary infection via the infected donor organ [5]. For these patients, the consequences of an active CMV infection include increased length of stay in hospital and higher medical costs, and it can result in graft-versus-host disease (GVHD), rejection of the transplanted organ, and death [6–8]. As a result, transplant recipients are monitored for CMV DNA via blood tests for prompt administration of treatment [9].

Currently available antiviral therapies for prophylaxis and treatment of CMV infection post transplant include ganciclovir, valganciclovir, foscarnet, cidofovir (not approved in Japan), and letermovir [3]. However, these therapies can exhibit severe side effects, such as bone marrow suppression or renal toxicities [3]. Maribavir is an oral benzimidazole nucleoside that inhibits viral kinase UL97 [10] and is active in vitro and in vivo against CMV, including strains resistant to ganciclovir, foscarnet, and/or cidofovir [11, 12]. Two phase III studies of maribavir have been conducted globally. In one of these studies, the phase III, randomized, open-label SOLSTICE trial, maribavir therapy was compared with investigator-assigned therapy (valganciclovir, ganciclovir, foscarnet, cidofovir) in hematopoietic cell transplant (HCT) and solid organ transplant (SOT) recipients with refractory CMV infection [13]. Maribavir was superior in achieving CMV viremia clearance at 8 weeks and in maintaining symptom control through 16 weeks. Fewer patients discontinued due to treatment-emergent adverse events (TEAEs) in the maribavir group (13.2% vs. 31.9%). In the other phase III study, the randomized, double-blind, non-inferiority AURORA trial, maribavir therapy was compared with valganciclovir therapy in patients with asymptomatic CMV infection post HCT [14]. The primary endpoint of confirmed CMV viremia clearance at week 8 (non-inferiority margin of maribavir of 7.0%) was not met. The key secondary endpoint showed 52.7% of patients treated with maribavir and 48.5% of those treated with valganciclovir maintained CMV viremia clearance without tissue-invasive disease at week 16. Compared with the valganciclovir group, fewer patients in the maribavir group discontinued due to TEAEs (27.8% vs. 41.2%). Despite the primary endpoint not being met, maribavir demonstrated comparable CMV viremia clearance during post-treatment follow-up. Currently, maribavir has been approved in more than 30 countries for the treatment of post-transplant CMV infection or disease that is refractory to treatment (with or without genotypic resistance) with ganciclovir, valganciclovir, cidofovir, or foscarnet [15, 16].

This article reports the first phase III clinical trial in Japan to assess the efficacy, safety, and pharmacokinetics (PK) of maribavir in Japanese hematopoietic stem cell transplant (HSCT) or SOT recipients with CMV infection. Patients with CMV infection who were defined as asymptomatic or refractory (with or without resistance) to ganciclovir, valganciclovir, or foscarnet were included in the study.

## Materials and methods

### Study design

**Fig. 1:**
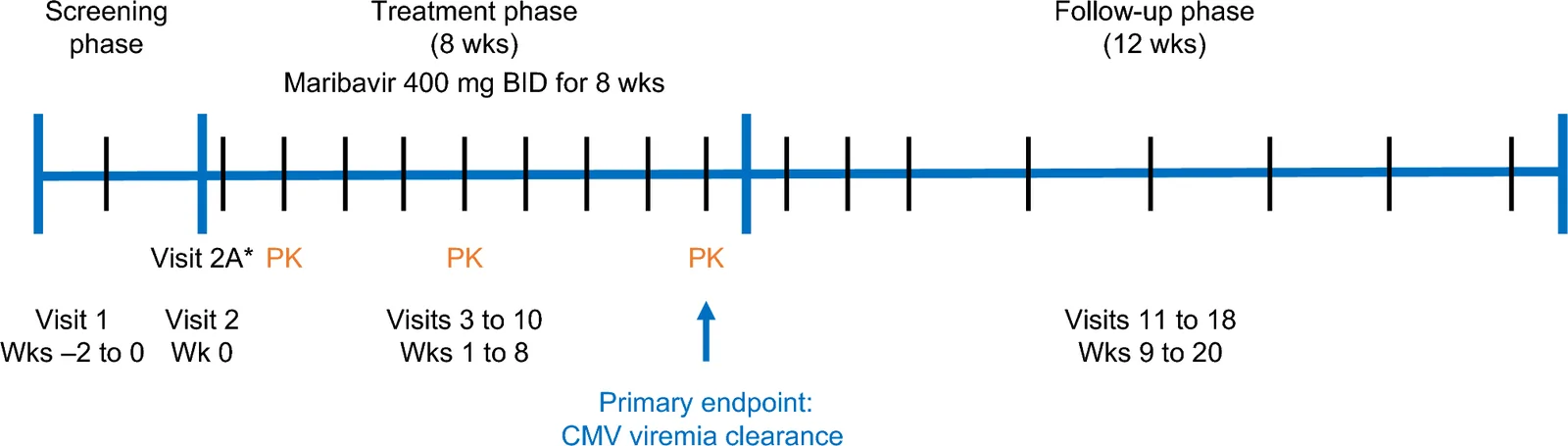
Study design schematic. *Visit 2A was required only for patients receiving tacrolimus, cyclosporine, or everolimus at baseline. *BID* Twice a day, *CMV* Cytomegalovirus, *PK* Pharmacokinetics, *Wks* Weeks

This phase III, multicenter, open-label, single-arm, interventional study in Japan consisted of a screening phase (weeks − 2 to 0), a treatment phase (weeks 1 to 8), and a follow-up phase (weeks 9 to 20) (Fig. 1). The study treatment phase consisted of clinic visits over 8 weeks, including assessment of graft outcomes and presence of adverse events. Study treatment was oral maribavir 400 mg, twice a day (BID) for 8 weeks. The recommended dose separation schedule was ≥ 8 h between daily doses. All patients who completed the study treatment phase through week 8 entered the 12-week follow-up phase. The follow-up phase consisted of weekly visits for the first 4 weeks (weeks 9 to 12), followed by visits every 2 weeks for the last 8 weeks (weeks 13 to 20).

For patients who, in the investigator’s judgment, had a lack of response or were unable to tolerate treatment and required discontinuation of study treatment, alternative anti-CMV treatment could be administered as deemed necessary. Patients who discontinued study treatment prior to week 8 completed the study assessments for the treatment phase.

These patients followed a modified schedule of assessments through the remaining weekly visits of the study treatment phase and a regular schedule of assessments through the 12-week follow-up phase.

The study protocol and amendments were approved by the institutional review board or independent ethics committee at each study site; the study period was January 18, 2022, to June 27, 2023. The study was conducted in accordance with the protocol, the International Council for Harmonisation (ICH) Guideline for Good Clinical Practice (GCP) E6 (ICH GCP, 1996; ICH E6 R2, 2016), and applicable national and local regulatory requirements. Written informed consent was provided by all patients or legal guardians before screening for inclusion in the study. The study was registered (ClinicalTrials.gov: NCT05137717; Japan Registry of Clinical Trials: jRCT2021210056).

### Study population

Patients were Japanese, aged ≥ 16 years, recipients of HSCT or SOT, and had a documented CMV infection with a screening value > 455 IU/mL in plasma in two consecutive assessments, separated by ≥ 1 day, as determined by quantitative polymerase chain reaction or comparable quantitative CMV DNA results from a central specialty laboratory. The screening value for CMV DNA was based on the threshold from the AURORA trial for asymptomatic patients [14]. Both samples were to be taken ≤ 14 days prior to first dose of study treatment, with the second sample obtained ≤ 5 days prior to first dose of study treatment. The current CMV infection (either primary or reactivation) was to be after HSCT or SOT, required treatment (in the investigator’s opinion), and was defined as asymptomatic, or resistant or refractory. Asymptomatic patients did not have CMV tissue-invasive disease or CMV syndrome (SOT patients only) at baseline [17]. Resistant or refractory patients had a current CMV infection that responded inadequately to the most recently administered of the anti-CMV treatment agent(s). In this study, refractory was defined as documented failure to achieve > 1 log_10_ decrease in plasma CMV DNA level after a ≥ 14-day treatment period with intravenous (IV) ganciclovir, oral valganciclovir, or IV foscarnet. Resistant was defined as documented ≥ 1 CMV genetic mutation associated with resistance to ganciclovir, valganciclovir, or foscarnet. Patients were excluded from the study if they had a diagnosis of central nervous system CMV tissue-invasive disease or CMV retinitis, were receiving valganciclovir, ganciclovir, foscarnet, or letermovir, or had a severe gastrointestinal illness within 24 h of the first study dose that would have precluded administration of oral medication.

### Outcome measures

The primary efficacy endpoints were confirmed clearance of plasma CMV DNA (CMV viremia clearance) at the end of week 8 for efficacy, and safety and tolerability of maribavir.

Confirmed viremia clearance was defined as plasma CMV DNA concentration below the lower limit of quantification (LLOQ) (i.e. < 34.5 IU/mL) in two consecutive post-baseline samples, separated by ≥ 5 days. CMV DNA concentrations were assessed by COBAS® 8800/COBAS® CMV Test (Roche Diagnostics, Mannheim, Germany) at a central specialty laboratory. CMV DNA blood tests and symptom assessments were conducted weekly up to week 12, and then biweekly up to week 20.

For the primary safety endpoints, TEAEs and treatment-emergent serious adverse events (SAEs) were collected for the on-treatment phase (time of study treatment initiation through 7 days after the last dose of study treatment, or until the non-study anti-CMV treatment initiation, whichever was earlier) and the overall study period (the time of the start of the study treatment through the end of the study). TEAEs were coded using Medical Dictionary for Regulatory Activities Version 26.0 by System Organ Class (SOC) and Preferred Term (PT), and assessed according to the National Cancer Institute Common Terminology Criteria for Adverse Events (NCI CTCAE) Version 5.0. Adverse events of special interest (AESIs) included taste disturbance (PTs: ageusia, dysgeusia, hypogeusia, and taste disorder) and neutropenia (PTs: agranulocytosis, febrile neutropenia, neutropenia, and neutrophil count decreased).

Secondary endpoints included maintenance of the confirmed CMV viremia clearance and infection symptom control achieved at week 8 and continued through weeks 12, 16, and 20; time to first confirmed viremia clearance at any time during the study; recurrence of confirmed CMV viremia requiring anti-CMV treatment during the 12-week follow-up period (among patients who had achieved clearance at week 8); recurrence of CMV viremia during study treatment and in the follow-up period after the patient had discontinued from study treatment; and confirmed CMV viremia clearance at a cut-off value of < 137 IU/mL at the end of week 8 (the cut-off value of < 137 IU/mL was the LLOQ in the SOLSTICE and AURORA trials [13, 14] and was only used in this secondary endpoint to compare the present and previous studies). Confirmed CMV viremia recurrence (referred to hereafter as “recurrence”) was defined as plasma CMV DNA concentration ≥ LLOQ in two consecutive plasma samples ≥ 5 days apart, after attaining confirmed viremia clearance. Mutations conferring resistance to maribavir were also assessed. Baseline and post-baseline plasma samples were tested by the central specialty laboratory to identify mutations in the viral UL97 and UL54 genes known to confer resistance to anti-CMV agents; UL27 was also tested. In addition, to characterize the PK profile of maribavir, predose plasma samples (before the morning dose) were obtained at weeks 1, 4, and 8 and summarized as minimum observed plasma concentration (C_min_). Plasma concentrations of maribavir were measured at Celerion Laboratories (Lincoln, NE, USA) using a validated liquid chromatography-tandem mass spectrometry (LLOQ = 0.200 μg/mL).

### Statistical analysis

The sample size was determined based on results from a feasibility assessment in Japan. Approximately 44 patients with asymptomatic CMV infection, and approximately three patients with resistant or refractory CMV infection, were expected to be enrolled.

The enrolled set consisted of all patients who signed an informed consent form and were involved in any study procedures. The full analysis set and the safety analysis set consisted of all patients who were administered ≥ 1 dose of maribavir and were used for the efficacy analyses and safety analyses, respectively. The PK set consisted of all patients in the safety set who had plasma samples drawn and tested for maribavir concentration.

Patients were grouped by CMV infection status (asymptomatic; resistant or refractory) for analyses. To be classified as a responder (confirmed clearance of plasma CMV DNA at the end of week 8), patients must have had two consecutive negative CMV DNA readings around week 8 (between day 39 and day 66, with one on day 53 or later) and no alternative anti-CMV DNA treatment prior to the first or second negative sample. Non-responders were patients who did not achieve plasma CMV DNA concentration < LLOQ, or those patients who received alternative non-study anti-CMV treatment before week 8 or had missing data to confirm response at week 8 due to early discontinuation. For subgroup analyses, efficacy analyses were performed by transplant type, presence of CMV syndrome, level of DNA viral load, presence of GVHD at baseline, age, sex, and CMV serostatus by donor (D+ or D−) and recipient (R+ or R−). All confidence intervals (CIs) were two-sided 95% exact CIs. SAS® 9.4 or higher (SAS Institute Inc., Cary, NC, USA) was used.

## Results

### Patients

**Fig. 2:**
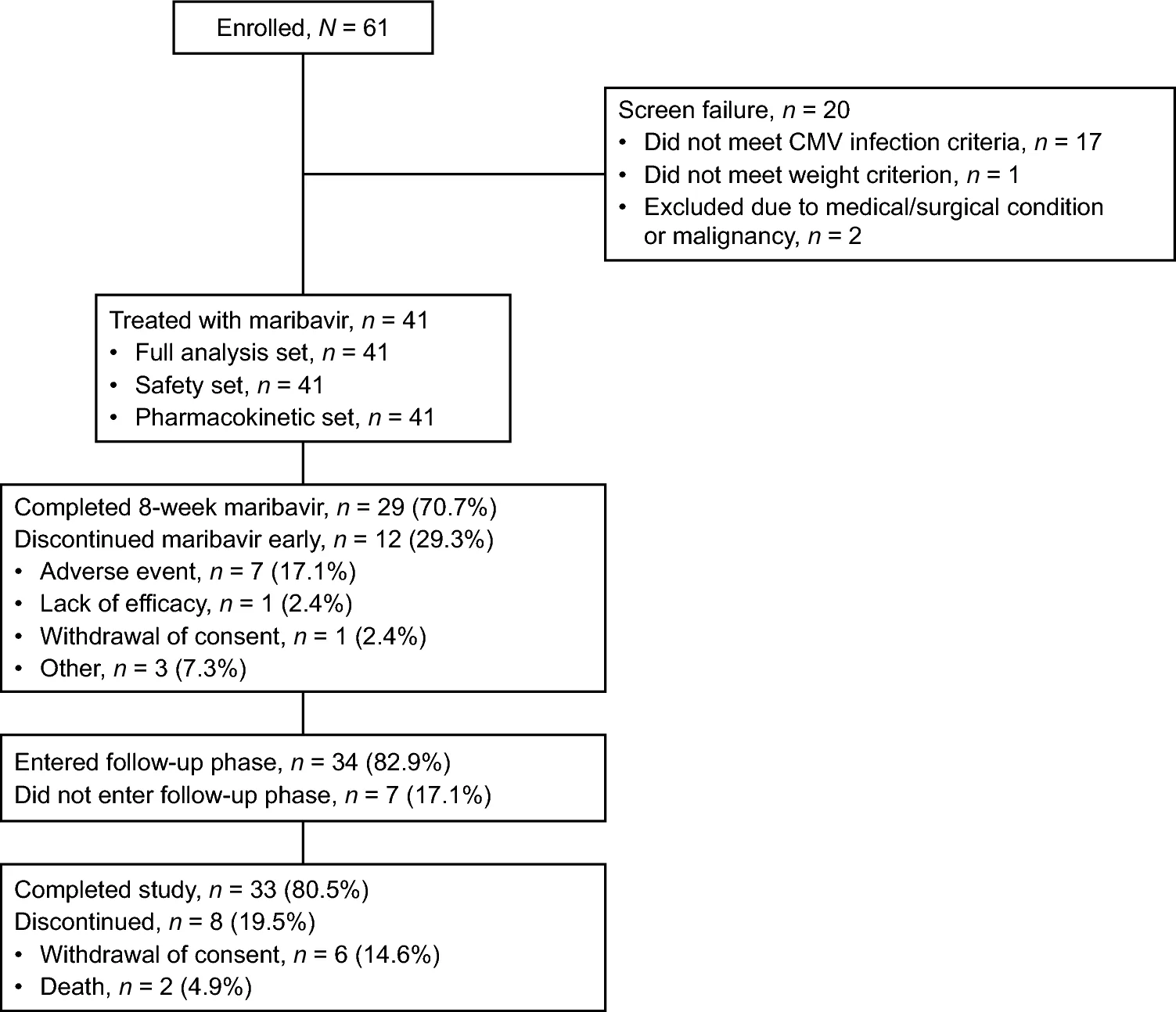
Patient disposition. *CMV* Cytomegalovirus

A total of 61 patients were enrolled across 22 sites; 20 of these patients were screen failures. Most screen failures (17 patients) were because of failure to meet the CMV infection criteria, while other screen failures were because of failure to meet the body weight criteria (≥ 40 kg) or meeting exclusion criteria. Forty-one patients were treated with ≥ 1 dose of maribavir and were included in all analysis sets (Fig. 2). A total of 29 patients (29/41, 70.7%) completed 8 weeks of study treatment, 34 patients (34/41, 82.9%) entered the follow-up phase, and 33 patients (33/41, 80.5%) completed the study. Reasons for early discontinuation of maribavir included TEAEs (7/41, 17.1%), lack of efficacy (1/41, 2.4%), withdrawal of consent (1/41, 2.4%), and other (3/41, 7.3%).

The median (quartile [Q]1, Q3) age was 56.0 (48.0, 63.0) years, weight was 55.80 (50.80, 61.80) kg, and body mass index was 21.04 (19.03, 22.91) kg/m^2^; 21 patients (21/41, 51.2%) were female (Table 1). Most patients (36/41, 87.8%) were HSCT recipients; in the SOT group, there were four kidney transplant recipients and one liver transplant recipient. Three patients were resistant or refractory to the most recent treatment for CMV. During the treatment phase, 35 patients (35/41, 85.4%) were prescribed concomitant immunosuppressants, and 31 patients (31/41, 75.6%) were prescribed corticosteroids for systemic use (Table 2).

**Table 1:** Demographic characteristics and resistance profile (full analysis set)

Variable*	All (*N* = 41)
Age (years), median (Q1, Q3)	56.0 (48.0, 63.0)
*Age categories (years)*	
< 18	0
18–44	7 (17.1)
45–64	27 (65.9)
≥ 65	7 (17.1)
Female	21 (51.2)
Height (cm), median (Q1, Q3)	163.80 (159.00, 171.40)
Weight (kg), median (Q1, Q3)	55.80 (50.80, 61.80)
BMI (kg/m^2^), median (Q1, Q3)	21.04 (19.03, 22.91)
*Current transplant type*	
SOT	5 (12.2)
Liver	1 (2.4)
Kidney	4 (9.8)
HSCT	36 (87.8)
*History of transplants prior to the current SOT transplant*	
No	5 (12.2)
Yes	0
*History of transplants prior to the current HSCT transplant*	
No	26 (63.4)
Yes	10 (24.2)
*Number of HSCT transplants prior to current treatment*	
0	26 (63.4)
1	10 (24.2)
*Underlying conditions for current HSCT transplantation*	
Acute myeloid leukemia	17 (41.5)
Chronic myeloid leukemia	1 (2.4)
Acute lymphocytic leukemia	3 (7.3)
Non-Hodgkin’s lymphoma	4 (9.8)
Myelodysplastic syndrome	8 (19.5)
Other myeloid malignancy	3 (7.3)
*Is this a recurrence of underlying disease*	
No	29 (70.7)
Yes	7 (17.1)
*Type of HSCT transplant*	
Autologous	2 (4.9)
Allogeneic	34 (82.9)
*If allogenic: Haploidentical*	
No	24 (58.5)
Yes	10 (24.4)
*HLA match type*	
Syngeneic (monozygotic twin)	0
HLA identical sibling (including non-monozygotic twin)	2 (4.9)
HLA matched other relative	0
HLA mismatched relative	9 (22.0)
Unrelated donor	23 (56.1)
*Stem cell source for current HSCT*	
Bone marrow	7 (17.1)
Peripheral blood stem cell	12 (29.3)
Cord blood	14 (34.1)
Other	1 (2.4)
*Current graft status at baseline for current HSCT*	
Partially engrafted	1 (2.4)
Functioning with complications	0
Functioning	33 (80.5)
*Type of preparative condition regimen for current HSCT*	
Myeloablative	16 (39.0)
Non-myeloablative	6 (14.6)
Reduced intensity conditioning regimen	11 (26.8)
Not applicable	1 (2.4)
*T-cell depletion modality*	
Ex vivo T-cell depletion	0
Alemtuzumab administration (in vivo)	0
Anti-thymocyte globulin administration (in vivo)	5 (12.2)
None	29 (70.7)
*Was a donor T-cell infusion given post HSCT*	
No	34 (82.9)
Yes	0
*CMV serostatus if SOT*^†^	
D+/R+	1 (20.0)
D−/R+	0
D+/R−	3 (60.0)
D−/R−	1 (20.0)
*CMV serostatus if HSCT*^†^	
D+/R+	14 (38.9)
D−/R+	12 (33.3)
D+/R−	4 (11.1)
D−/R−	3 (8.3)
Missing	3 (8.3)
*First episode of post-transplant CMV infection*	
No	13 (31.7)
Yes	28 (68.3)
*History of tissue-invasive CMV disease*	
No	38 (92.7)
Yes	3 (7.3)
*Type of prior tissue-invasive CMV disease*	
Colitis	2 (4.9)
Enteritis	1 (2.4)
*Resistance mutation by central laboratory result*	
No	41 (100.0)
Yes	0
*Acute GVHD status at baseline*	
Presence	8 (19.5)
Absence	33 (80.5)
*Chronic GVHD confirmed*	
Presence	4 (9.8)
Absence	37 (90.2)
*Baseline total leukocyte count (10*^*9*^*/L)*	
Mean (SD)	6.08 (3.396)
Median	5.00
Q1, Q3	3.90, 7.70
Range	1.2‒19.4
*Categorization of current CMV infection*	
CMV syndrome (SOT patients only)	0
CMV tissue-invasive disease	2 (4.9)
Asymptomatic tissue-invasive disease	39 (95.1)
*Current CMV infection*	
Asymptomatic	38 (92.7)
Resistant or refractory	3 (7.3)
*CMV DNA category based on central laboratory results*	
High (≥ 91,000 IU/mL)	0
Intermediate (≥ 9100 and < 91,000 IU/mL)	13 (31.7)
Low (< 9100 IU/mL)	28 (68.3)
*Baseline CMV DNA levels from central laboratory (IU/mL)*	
Median	2200.000
Q1, Q3	712.000, 14,700.000
Range	17.25‒72,700.00
*Days from onset of current CMV infection to first dose of study treatment*	
Median	15.0
Q1, Q3	10.0, 22.0
Range	4‒71
*Days from current transplant to the first dose of study treatment*	
Median	100.0
Q1, Q3	68.0, 147.0
Range	28‒774
*Days from the current transplant to the onset of the current CMV infection*	
Median	70.0
Q1, Q3	38.0, 138.0
Range	7‒756

Data are *n* (%) unless stated otherwise. Percentages are based on the patients in the full analysis set unless specified otherwise

*BMI* Body mass index, *CMV* Cytomegalovirus, *D* Donor, *GVHD* Graft-versus-host disease, *HLA* Human leukocyte antigen, *HSCT* Hematopoietic stem cell transplant, *Q1* Quartile 1, *Q3* Quartile 3, *R* Recipient, *SD* Standard deviation, *SOT* Solid organ transplant

^*^Demographic and baseline characteristics were determined using the screening visit or last observation on or prior to first dose of study treatment, whichever was later

^†^Percentages were based on the number of patients within the category indicated

**Table 2:** Immunosuppressants and corticosteroids for systemic use during the treatment phase

Therapeutic class Preferred drug name, *n* (%)	All (*N* = 41)
Immunosuppressants	35 (85.4)
Tacrolimus	27 (65.9)
Ciclosporin	8 (19.5)
Mycophenolic acid	8 (19.5)
Corticosteroids for systemic use	31 (75.6)
Prednisolone	25 (61.0)
Hydrocortisone	7 (17.1)
Methylprednisolone	4 (9.8)
Dexamethasone	1 (2.4)
Prednisone	1 (2.4)

Percentages are based on the number of patients in the safety analysis set, and medications were coded using the WHO Drug Dictionary dated March 2021

Listed immunosuppressants and corticosteroids for systemic use are those with a start date prior to the date of the first dose of study treatment and continuing after the first dose of study treatment or with a start date and time after the study treatment initiation and before the end of the on-treatment period

The on-treatment period starts at the time of study treatment initiation through 7 days after the last dose of study treatment. For patients who transferred from the study treatment to a non-study anti-CMV treatment, the on-treatment period started at the time of the study treatment initiation through 7 days after the last dose of study treatment, or until the non-study anti-CMV treatment initiation, whichever was earlier

*CMV* Cytomegalovirus, *WHO* World Health Organization

### Primary outcome measure

A total of 28 patients (28/41, 68.3%) achieved confirmed CMV viremia clearance at week 8 (Table 3). This included 27 patients (27/38, 71.1%) with asymptomatic CMV and one patient (1/3, 33.3%) with refractory CMV. Confirmed CMV viremia clearance responses by patient baseline characteristics are reported in Table 4. CMV viremia clearance at week 8 was achieved for two SOT recipients (2/5, 40.0%) and 26 HSCT recipients (26/36, 72.2%).

**Table 3:** Confirmed CMV viremia clearance at week 8 (full analysis set)

Group *n* (%)	All (*N* = 41)	Asymptomatic (*N* = 38)	Resistant or refractory (*N* = 3)
Responders	28 (68.3)	27 (71.1)	1 (33.3)
Non-responders	13 (31.7)	11 (28.9)	2 (66.7)
Proportion of responders	68.3	71.1	33.3
(95% exact CI)	(51.9–81.9)	(54.1–84.6)	(0.8–90.6)

*CI* Confidence interval; *CMV* Cytomegalovirus

**Table 4:** Subgroup analyses of confirmed CMV viremia clearance response (full analysis set)

Baseline characteristic type	*n*/*N*	% (95% exact CI)
*Transplant type*		
SOT	2/5	40.0 (5.3–85.3)
HSCT	26/36	72.2 (54.8–85.8)
*If SOT, organ type*		
Kidney	2/4	50.0 (6.8–93.2)
Liver	0/1	0
*CMV syndrome (SOT) or CMV tissue-invasive disease*		
Yes	0/2	0
No	28/39	71.8 (55.1–85.0)
*CMV DNA viral load*		
Low*	19/28	67.9 (47.6–84.1)
Intermediate^†^	9/13	69.2 (38.6–90.9)
*Acute GVHD at baseline*		
Absent	22/33	66.7 (48.2–82.0)
Present	6/8	75.0 (34.9–96.8)
*Age category (years)*		
18–44	4/7	57.1 (18.4–90.1)
45–64	19/27	70.4 (49.8–86.2)
≥ 65	5/7	71.4 (29.0–96.3)
*Sex*		
Male	12/20	60.0 (36.1–80.9)
Female	16/21	76.2 (52.8–91.8)
*CMV serostatus*		
D+/R−	4/7	57.1 (18.4–90.1)
D+/R+	12/15	80.0 (51.9–95.7)
D−/R+	8/12	66.7 (34.9–90.1)
D−/R−	3/4	75.0 (19.4–99.4)
Missing	1/3	33.3 (0.8–90.6)

*CI* Confidence interval, *CMV* Cytomegalovirus, *D* Donor, *GVHD* Graft-versus-host disease, *HSCT* Hematopoietic stem cell transplant, *n* Number of responders at study week 8, *N* Number of patients in each subgroup, *R* Recipient, *SOT* Solid organ transplant

^*^CMV DNA concentration < 9100 IU/mL

^†^CMV DNA concentration ≥ 9100 and < 91,000 IU/mL

### Secondary outcome measures

**Fig. 3:**
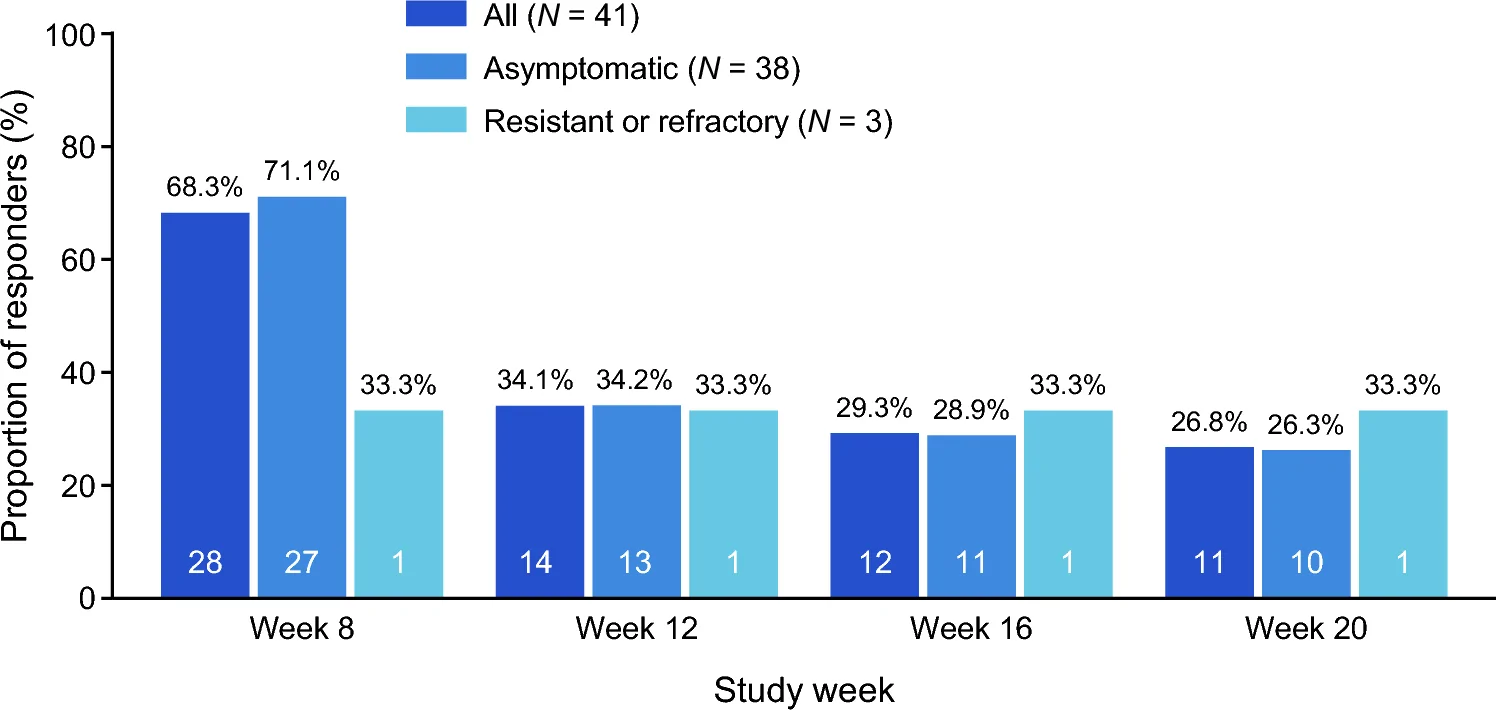
Confirmed CMV viremia clearance response at study week 8 followed by maintenance through study weeks 12, 16, and 20. *CMV* Cytomegalovirus

The number of patients in the full analysis set who achieved CMV viremia clearance at week 8 and maintained viremia clearance off treatment through weeks 12, 16, and 20 was 14 (14/41, 34.1%), 12 (12/41, 29.3%), and 11 (11/41, 26.8%), respectively (Fig. 3). For patients who had achieved CMV viremia clearance at week 8, recurrence of CMV viremia requiring treatment during the 12-week follow-up period was observed for 12 patients (12/28, 42.9%). Among these 12 patients, seven patients were cord blood transplantation recipients. Only one of these patients experienced an increase in immunosuppressant dose between achievement of CMV viremia clearance and its recurrence.

The observed median time to first CMV viremia clearance was 19.5 days (range 8 to 38 days) (Table 5). Overall, after receiving maribavir, 36 patients (36/41, 87.8%) achieved first confirmed CMV viremia clearance at any time during the study, all of which were by week 8. Recurrence of CMV viremia at any time during the study was observed for 21 patients (21/36, 58.3%); five patients (5/36, 13.9%) experienced recurrence during the treatment phase (three patients on study treatment), and 16 patients (16/36, 44.4%) during the follow-up phase. Overall, 18 patients (18/36, 50.0%) experienced recurrence while off study treatment. The number of patients who achieved CMV viremia clearance with a cut-off value of < 137 IU/mL at week 8 was 30 patients (30/41, 73.2%), including 29 patients (29/38, 76.3%) with asymptomatic CMV infection and one patient (1/3, 33%) with resistant or refractory CMV infection.

**Table 5:** Time to first confirmed CMV viremia clearance at any time on study

	All (*N* = 41)	Asymptomatic (*N* = 38)	Resistant or refractory (*N* = 3)
Number of patients with first confirmed CMV viremia clearance within week 8	36 (87.8)	34 (89.5)	2 (66.7)
Number of patients with first confirmed CMV viremia clearance at any time on study	36 (87.8)	34 (89.5)	2 (66.7)
Number of patients censored*	5 (12.2)	4 (10.5)	1 (33.3)
Observed event time for those who had confirmed CMV viremia clearance (days), median (range)	19.5 (8‒38)	19.5 (8‒35)	22.0 (15‒29)

Data are *n* (%) unless stated otherwise

*CMV* Cytomegalovirus

^*^Patients who did not achieve CMV viremia clearance were censored on the date of last CMV DNA assessment on study prior to the initiation of alternative anti-CMV medication

Overall, five patients (5/41, 12.2%) developed resistance to maribavir as identified via resistance-associated amino acid substitutions (RAS), all of which have been described previously [14, 18]; one patient achieved viremia clearance at week 8, and 2/3 patients who switched to alternative anti-CMV treatment were successful in achieving viremia clearance.

**Fig. 4:**
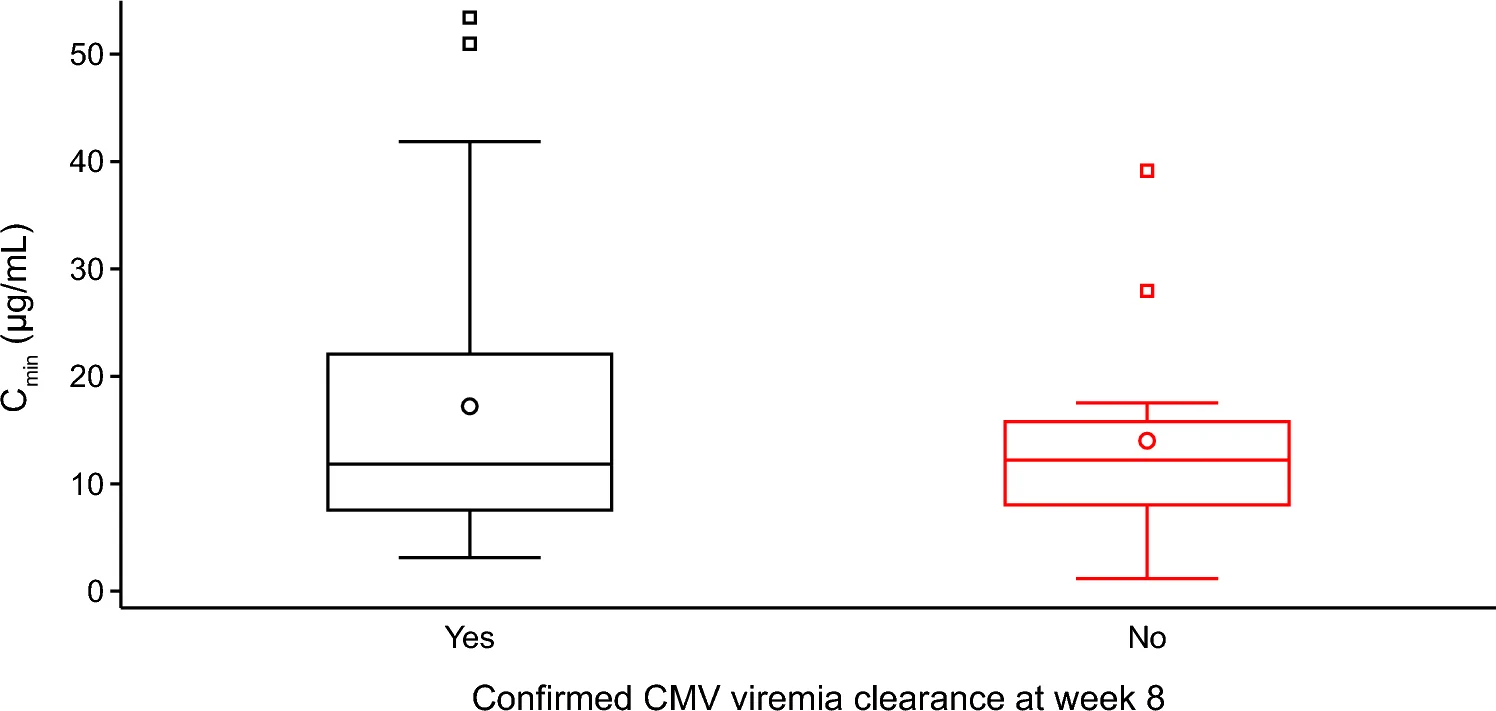
Box plot of average maribavir C_min_ by confirmed CMV viremia clearance response at week 8 (pharmacokinetic set; *N* = 41). C_min_ values from week 1 to week 8 were averaged. The bottom and top box lines represent the lower and upper quartiles (Q1 and Q3), respectively, with the median represented by the middle box line. Bottom whisker represents the smallest value inside Q1–1.5 × IQR. Top whisker represents the largest value inside Q3 + 1.5 × IQR. The means are represented by circle markers and the outliers are represented by square markers. *C*_*min*_ minimum observed plasma concentration, *CMV* Cytomegalovirus, *IQR* Interquartile range (Q3–Q1), *Q1* Quartile 1, *Q3* Quartile 3

Following repeated oral administration of maribavir 400 mg BID, mean C_min_ at weeks 1, 4, and 8 was 14.28, 18.07, and 16.66 μg/mL, respectively (Table 6). There were no data below the LLOQ. The percentage coefficient of variation (%CV) of C_min_ at each time point ranged from 84.99 to 100.65, suggesting large variability of C_min_. Although no statistical testing was performed, there was no apparent relationship between C_min_ (median [range] µg/mL: responders, 11.83 [3.1 to 53.4]; non-responders, 12.21 [1.2 to 39.2]) and CMV viremia clearance at week 8 (Fig. 4).

**Table 6:** Predose plasma maribavir concentrations (pharmacokinetic set)

C_min_ (μg/mL)	Week 1 (*n* = 36)	Week 4 (*n* = 31)	Week 8 (*n* = 29)
Mean (SD)	14.28 (12.602)	18.07 (18.190)	16.66 (14.159)
%CV	88.26	100.65	84.99
Median	10.26	13.10	13.10
Range	1.18‒55.10	2.89‒76.60	2.12‒61.40
Geometric mean	10.08	12.31	12.37
Geometric %CV	105.73	108.05	95.26

*C*_*min*_ Minimum observed plasma concentration, *CV* Coefficient of variation, *SD* Standard deviation

### Safety and tolerability measures

Median (range) duration of exposure to study treatment was 56 (2 to 59) days. During the on-treatment phase, 36 patients (36/41, 87.8%) had ≥ 1 TEAE (Table 7) and 15 patients (15/41, 36.6%) had TEAEs considered related to study treatment by the investigator (Table 8). The most common TEAEs by SOC were nervous system disorders (16/41, 39.0%), followed by gastrointestinal disorders, and general disorders and administration site conditions (14/41, 34.1% each) (Table 7). The most common TEAEs by PT were nausea (10/41, 24.2%), anemia (7/41, 17.1%), pyrexia (6/41, 14.6%), and headache (6/41, 14.6%). Nine patients (9/41, 22.0%) reported TEAEs leading to study treatment discontinuation.

**Table 7:** TEAEs that occurred in ≥ 5% of patients during the on-treatment phase* (safety analysis set)

System Organ Class Preferred Term	*n* (%) (*N* = 41)	Number of events
Any TEAE	36 (87.8)	122
*Blood and lymphatic system disorders*	10 (24.2)	12
Anemia	7 (17.1)	8
*Gastrointestinal disorders*	14 (34.1)	21
Nausea	10 (24.2)	10
*General disorders and administration site conditions*	14 (34.1)	15
Pyrexia	6 (14.6)	6
Generalized edema	4 (9.8)	4
Edema peripheral	3 (7.3)	3
*Metabolism and nutrition disorders*	10 (24.4)	12
Decreased appetite	5 (12.2)	5
*Nervous system disorders*	16 (39.0)	16
Headache	6 (14.6)	6
Taste disorders	4 (9.8)	4
*Vascular disorders*	4 (9.8)	4
Hypertension	3 (7.3)	3

Patients were counted once per System Organ Class and once per Preferred Term per treatment

*CMV* Cytomegalovirus, *n* Number of patients experiencing the event, *N* Number of patients, *TEAE* Treatment-emergent adverse event

^*^The on-treatment phase started at the time of study treatment initiation through 7 days after the last dose of study drug, or until the non-study CMV treatment initiation, whichever was earlier

**Table 8:** TEAEs related to study treatment that occurred during the on-treatment phase* (safety analysis set)

System Organ Class Preferred Term	All (*N* = 41) *n* (%), m	Asymptomatic (*N* = 38) *n* (%), m	Resistant or refractory (*N* = 3) *n* (%), m
Any related TEAE	15 (36.6), 30	12 (31.6), 25	3 (100.0), 5
*Blood and lymphatic system disorders*	4 (9.8), 5	3 (7.9), 4	1 (33.3), 1
Anemia	1 (2.4), 2	1 (2.6), 2	0
Cytopenia	1 (2.4), 1	0	1 (33.3), 1
Febrile neutropenia	1 (2.4), 1	1 (2.6), 1	0
Thrombotic microangiopathy	1 (2.4), 1	1 (2.6), 1	0
*Gastrointestinal disorders*	6 (14.6), 8	5 (13.2), 7	1 (33.3), 1
Abdominal pain	1 (2.4), 1	1 (2.6), 1	0
Nausea	6 (14.6), 6	5 (13.2), 5	1 (33.3), 1
Vomiting	1 (2.4), 1	1 (2.6), 1	0
*General disorders/administration site conditions*	3 (7.3), 3	2 (5.3), 2	1 (33.3), 1
Face Edema	1 (2.4), 1	0	1 (33.3), 1
Edema peripheral	1 (2.4), 1	1 (2.6), 1	0
Pyrexia	1 (2.4), 1	1 (2.6), 1	0
*Hepatobiliary disorders*	2 (4.9), 2	1 (2.6), 1	1 (33.3), 1
Hepatic failure	1 (2.4), 1	0	1 (33.3), 1
Hyperbilirubinemia	1 (2.4), 1	1 (2.6), 1	0
*Investigations*	2 (4.9), 4	1 (2.6), 3	1 (33.3), 1
Hepatic enzyme increased	1 (2.4), 1	1 (2.6), 1	0
Immunosuppressant drug level increased	1 (2.4), 1	0	1 (33.3), 1
Neutrophil count decreased	1 (2.4), 2	1 (2.6), 2	0
*Metabolism and nutrition disorders*	2 (4.9), 2	2 (5.3), 2	0
Decreased appetite	1 (2.4), 1	1 (2.6), 1	0
Hypomagnesemia	1 (2.4), 1	1 (2.6), 1	0
*Nervous system disorders*	5 (12.2), 5	5 (13.2), 5	0
Dysgeusia	2 (4.9), 2	2 (5.3), 2	0
Headache	2 (4.9), 2	2 (5.3), 2	0
*Taste disorder*	1 (2.4), 1	1 (2.6), 1	0
Vascular disorders	1 (2.4), 1	1 (2.6), 1	0
Hypertension	1 (2.4), 1	1 (2.6), 1	0

If ≥ 1 TEAE occurred with the same Preferred Term for the same patient, the patient was counted only once for that Preferred Term using the most severe and most related occurrence for the summarization by severity and by relationship to study treatment

*m* Number of events, *n* Number of patients experiencing the event, *N* Number of patients, *TEAE* Treatment-emergent adverse event

^*^The on-treatment period started at the time of study treatment initiation through 7 days after the last dose of study

For AESIs (on-treatment phase), taste disturbance occurred in six patients (6/41, 14.6%) and presented as taste disorder (four patients; all events were NCI CTCAE grade 1) and dysgeusia (two patients; all events grade 1). Neutropenia occurred in four patients (4/41, 9.8%; including two patients [2/41, 4.9%] with treatment-related neutropenia) and included neutrophil count decreased (two patients; grades 2 and 3), febrile neutropenia (one patient; grade 3), and neutropenia (one patient; grade 4). Tissue-invasive CMV disease (CMV chorioretinitis) was observed in one patient (1/41, 2.4%); however, the event was considered not related to the study drug because chorioretinitis occurred before the start of study treatment. No central nervous system CMV tissue-invasive disease occurred. Renal disorder (PT: prerenal failure) was experienced by one patient (1/41, 2.4%).

Four patients (4/41, 9.8%) had treatment-related TEAEs that led to maribavir discontinuation; these were febrile neutropenia, thrombotic microangiopathy, decreased appetite, and headache. Of these, febrile neutropenia in one patient was reported as a treatment-related SAE by the investigator. A second treatment-related SAE (hyperbilirubinemia) was reported in one patient. Two patients (2/41, 4.9%) died, one patient (1/41, 2.4%) had GVHD (on-treatment phase), and one patient (1/41, 2.4%) had transplant complications (overall study period). These deaths were deemed unrelated to maribavir by the investigator.

## Discussion

This was the first phase III clinical trial in Japan to evaluate the efficacy and safety of maribavir in Japanese HSCT or SOT recipients with CMV infection, including patients with CMV infection who were resistant or refractory to ganciclovir, valganciclovir, or foscarnet. Maribavir exhibited comparable efficacy to previous studies, with 28 patients (28/41, 68.3%) achieving CMV viremia clearance at week 8 of treatment [13, 14]. Overall, 36 patients (36/41, 87.8%) achieved CMV viremia clearance at any time during the study, all of which were by week 8. Regarding PK, maribavir appeared to have reached steady state by week 1, consistent with existing PK data from non-Japanese populations [19, 20], and a large variability in C_min_ was observed, consistent with existing population PK model analysis results for maribavir [21, 22]. There was no apparent relationship between C_min_ and CMV viremia clearance at week 8. Maribavir was generally well tolerated, and no new safety signals were identified.

For resistant or refractory patients, one patient (1/3, 33.3%) in this study met the primary efficacy endpoint of CMV viremia clearance at week 8, which was relatively lower than the larger international phase III clinical trial, SOLSTICE, where the efficacy rate was 55.7% (131/235) [13]. This difference may be attributed to the very limited number of refractory CMV patients (*N* = 3) in this study compared with SOLSTICE (*N* = 235) [13]. For asymptomatic patients, 27 patients (27/38, 71.1%) in this study met the primary efficacy endpoint of CMV viremia clearance at week 8, which was comparable to the larger international phase III clinical trial, AURORA, where the efficacy rate was 69.6% (190/273) [14]. Furthermore, the overall rate of treatment-emergent RAS to maribavir in the present study was within the same range as in AURORA (24/273, 8.8%) and SOLSTICE (60/234, 26%) [14, 18]. Considering that those who develop maribavir resistance tend not to achieve CMV viremia clearance, and that alternative anti-CMV treatment often works under such situations, clinicians should ensure CMV DNA levels are monitored and switch treatments in the case of inadequate response.

In the current study, maribavir was well tolerated, safety results were in line with previously published maribavir trials [13, 14], and no new safety signals emerged. Although two patients (2/41, 4.9%) died during the study period, these deaths were not considered related to maribavir. Most patients (36/41, 87.8%) experienced TEAEs, which was similar to SOLSTICE (228/234, 97.4%) and AURORA (268/273, 98.2%) [13, 14]. Overall, nine patients (9/41, 22%) discontinued study treatment due to TEAEs, while 13.2% (31/234) and 27.8% (76/273) of patients discontinued due to TEAEs in SOLSTICE [13] and in AURORA [14], respectively. A total of six patients (6/41, 14.6%) reported taste disturbance TEAEs (taste disorder and dysgeusia), which were transient and not treatment limiting. Dysgeusia was reported in 37.2% (87/234) and 17.2% (47/273) of patients in SOLSTICE and AURORA, respectively [13, 14]. Four patients (4/41, 9.8%) reported neutropenia (as an AESI category), with one patient experiencing febrile neutropenia, which was considered a treatment-related SAE. Renal disorder was reported for one patient (1/41, 2.4%), which was mild in severity and considered not related to study treatment. Although there were only a small number of resistant or refractory patients in this study, TEAEs were similar to the overall cohort, suggesting that these patients did not have apparent safety concerns compared with asymptomatic patients.

A strength of this prospective multicenter trial was the inclusion of both HSCT and SOT recipients, and all serotype combinations were eligible. Biochemical tests for key study endpoints were performed at a central specialty laboratory to ensure quality control, optimization, and consistency between samples.

The main limitations of this study were that there was no control group, the overall sample size was small, the number of resistant or refractory patients was limited, and only Japanese HSCT or SOT recipients were included. It is noted that systemic use of corticosteroids may have an impact on antiviral therapy [23]; however, due to limited data on the use of concomitant corticosteroids in this study, it was not possible to evaluate their impact. The fixed treatment duration of 8 weeks in this study may not fully align with clinical practice or patient needs. Trials where administration time is adjusted to reflect clinical practice for the target patient population will provide more information. In addition, because this study lacked a control group and had a small sample size, it was difficult to assess the cost-effectiveness of maribavir in the Japanese healthcare setting. Studies that better predict and understand CMV recurrence and examine the effectiveness and safety of maribavir in the real world will also be informative.

In conclusion, maribavir was effective in achieving CMV viremia clearance, with an acceptable safety and tolerability profile, in Japanese HSCT or SOT recipients with CMV infection, including patients with inadequate response to prior anti-CMV therapies.

## Data Availability

The datasets, including the redacted study protocol, redacted statistical analysis plan, and individual participants data supporting the results reported in this article, will be made available within three months from initial request, to researchers who provide a methodologically sound proposal. The data will be provided after its de-identification, in compliance with applicable privacy laws, data protection and requirements for consent and anonymization.

## Abbreviations

AESI: Adverse event of special interest
BID: Twice a day
CI: Confidence interval
C_min_: Minimum observed plasma concentration
CMV: Cytomegalovirus
%CV: Percent coefficient of variation
D: Donor
GCP: Good Clinical Practice
GVHD: Graft-versus-host disease
HCT: Hematopoietic cell transplant
HSCT: Hematopoietic stem cell transplant
ICH: International Council for Harmonisation
IV: Intravenous
LLOQ: Lower limit of quantification
PK: Pharmacokinetics
PT: Preferred Term
Q: Quartile
R: Recipient
RAS: Resistance-associated amino acid substitution
SAE: Serious adverse event
SOC: System Organ Class
SOT: Solid organ transplant
TEAE: Treatment-emergent adverse event
